# An integrated approach for the systematic identification and characterization of heart-enriched genes with unknown functions

**DOI:** 10.1186/1471-2164-10-100

**Published:** 2009-03-06

**Authors:** Shizuka Uchida, André Schneider, Marion Wiesnet, Benno Jungblut, Polina Zarjitskaya, Katharina Jenniches, Karsten grosse Kreymborg, Werner Seeger, Thomas Braun

**Affiliations:** 1 Max-Planck-Institute for Heart and Lung Research, Parkstr. 1, 61231 Bad Nauheim, Germany; 2 University of Giessen Lung Center (UGLC), Medical Clinic II and V, Justus-Liebig-University Giessen, Klinikstrasse 36, 35392 Giessen, Germany

## Abstract

**Background:**

High throughput techniques have generated a huge set of biological data, which are deposited in various databases. Efficient exploitation of these databases is often hampered by a lack of appropriate tools, which allow easy and reliable identification of genes that miss functional characterization but are correlated with specific biological conditions (e.g. organotypic expression).

**Results:**

We have developed a simple algorithm (DGSA = Database-dependent Gene Selection and Analysis) to identify genes with unknown functions involved in organ development concentrating on the heart. Using our approach, we identified a large number of yet uncharacterized genes, which are expressed during heart development. An initial functional characterization of genes by loss-of-function analysis employing morpholino injections into zebrafish embryos disclosed severe developmental defects indicating a decisive function of selected genes for developmental processes.

**Conclusion:**

We conclude that DGSA is a versatile tool for database mining allowing efficient selection of uncharacterized genes for functional analysis.

## Background

In recent years, the advent of high-throughput analytical techniques, such as microarrays and serial analysis of gene expression (SAGE), has led to a rapid accumulation of biological data. The large size of databases, which are now within the petabytes range [1] precludes manual analysis and renders unsystematic approaches obsolete. To cope with these new challenges and to facilitate efficient data analyses, numerous academic and commercial software packages and databases have been developed [2-5]. Yet, genes to which no biological function has been assigned compromise the usability of these data. In particular, the construction of linear and non-linear models has proven to be difficult if "function unknown genes" are included [6]. Any attempts to integrate and analyze complex biological data from various "omics" techniques (e.g. transcriptomics, proteomics, and metabolomics) to understand biological phenomena as a collection of interconnected systems will depend on the functional annotation of the majority of their components [7]. Thus, successful approaches to "Systems Biology" will depend on the identification and functional characterization of most if not all players in the system under study [8,9]. An obvious, almost trivial conclusion is the systematic identification of only those genes, which lack biologically valid annotations, followed by functional characterization. Surprisingly, only few attempts were made to address this problem systematically although most databases contain large numbers of genes, which have only been identified by computational sequence analyses [10,11]. Instead, most efforts have concentrated on large-scale functional analyses such a proteome-wide protein interaction screens [12,13] or genome-wide siRNA knockdowns [14-16], which are much more difficult to perform and are notoriously unreliable [17-19]. Other approaches such as ChIP-on-chip [20] and ChIP-Seq [21] are limited to specific biological properties (e.g. DNA binding).

The relevance of this problem becomes apparent in experiments that use large datasets such as in DNA microarray hybridizations. Transcriptional profiling of a diseased heart, for example, will identify changes in the expression of known signaling pathways as well as genes, which do not fit into existing regulatory circuits (see reviews [22]). Although the expression profile of the latter group of genes might correlate strongly with certain physiological conditions or developmental status suggesting potential interactions with known regulatory networks, reliable data to describe the biological function are most often missing.

Here, we describe a strategy for the systematic identification of genes, which are correlated with a specific biological condition (e.g. organ-specific expression) but lack functional characterization. Our approach is based on the systematic exploitation of existing databases such as the UniGene database [23], which contain information about organ-specific expression patterns, applying a number of different filters. Information gathered from expression databases were matched with publication records based on the assumption that virtually all genes with a functional characterization will also relay to a corresponding publication. Although the focus of the study was on "heart-enriched genes", our approach is applicable to virtually all organs and physiological status. The expression profiles of identified gene were validated by RT-PCR using different murine adult organs and by whole-mount *in situ *(WISH) hybridization of mouse embryos. The functional role of selected genes was also approached by morpholino knock-down experiments in zebrafish embryos

## Results

### In Silico Screening of UniGene Database

**Figure 1:**
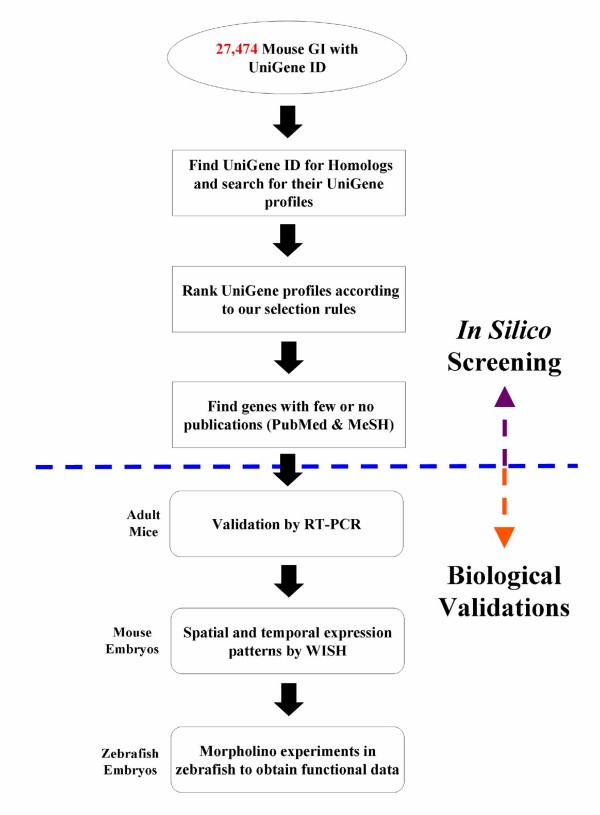
**Flowchart of in silico UniGene profile screening**. At the time of this study, 27,474 mouse GeneIDs (GI) with UniGene IDs were analyzed.

Several public databases for "omics" data are available, which cover the expression profile of individual genes in various organisms to a different extent. For our study, we employed the UniGene database, which contains comprehensive coverage of various organisms and a collection of expression data. Each UniGene ID has an attached "Expression Profile", which can be used to search for specific expression sites. To identify genes in a systematic manner, which show a preferential expression in the heart but lack a proper functional annotation, we selected 4 different organisms for our analysis (rat, mouse, human, and chicken) (Figure 1). Applying the rule "Select genes whose rank for 'heart' is under the top 20% across homologues" resulted in the identification of 2,348 mouse genes, which fitted the rule in more than one organism. It should be pointed out that the combination of expression data from different organisms reduced the number of false-positive candidates. Since the expression profile of genes is normally conserved among evolutionary close species, any expression profile that is unique for a single species is likely to indicate faulty expression profiling rather than a biological meaningful result.

**Figure 2:**
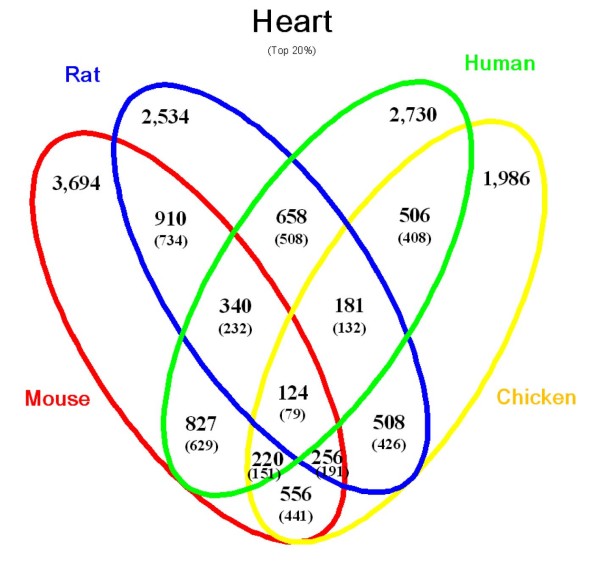
**Venn diagram for heart-enriched genes**. The numbers of heart-enriched genes selected for the top 20% in the ranking of UniGene tissue expression profiles are shown for the 4 organisms analyzed and displayed as combinations of these organisms. In each combination, the number of genes with less than 2 articles after MeSH term filtering for "heart" is shown in parenthesis.

After identification of heart-enriched genes, we searched for genes in this group, which lack a proper annotation. We assumed that genes with no or very few publications are "uncharacterized genes". To avoid papers describing large screening efforts (e.g. microarrays, sequencing), which usually do not include a functional analysis, we excluded papers that contained more than 100 GeneIDs (GI). Obviously, this selection is rather strict resulting in exclusion of potentially interesting genes. To address this problem, we added another selection criterion that was based on Medical Subject Headings (MeSH). Inclusion of the MeSH term for "heart" with less than 2 publications resulted in the identification of 1,975 "uncharacterized" mouse genes, which show an expression in the heart. Figure 2 depicts the numbers of heart-enriched genes, which were identified using different combinations of organisms. Selection of genes based on the concurrent expression in the heart in *all *model organisms massively decreased the number of positive genes yielding 124 heart-enriched genes of which 79 are uncharacterized. Unfortunately, the quality of the expression data is not identical in all model organisms, which resulted in exclusion of genes, which have not been properly accessed but in fact are expressed in the heart. An improvement of the quality of the expression data will reduce the number of false-negative genes in the future.

### Validation of the In Silico Screening

To demonstrate the efficiency of our method, we searched the Gene Ontology project [24] for the term "heart" and matched the results with our data set that was based on the analysis of the expression data including genes, which lack an annotation. We used 79 GO terms that contain the phrase "heart" and screened the gene names and symbols of the 4 selected model organisms. We found that 32 out of the 79 GO terms contained at least one gene in the organisms studied. The number of genes that were obtained through our method and the coverage of GO terms in percentages of the genes that matched our selection criteria are shown in Additional File 1. For example, about 30% of the genes that carry the GO term "heart development (GO:0007507)" were covered by our method (34% for mouse, 28% for rat, 37% for human, and 37% for chicken). The information about all 43 mouse genes that were extracted through our method using the GO term "heart development" is listed in Additional File 2.

### Further Selection Rules to Eliminate Ubiquitously Expressed Genes

**Figure 3:**
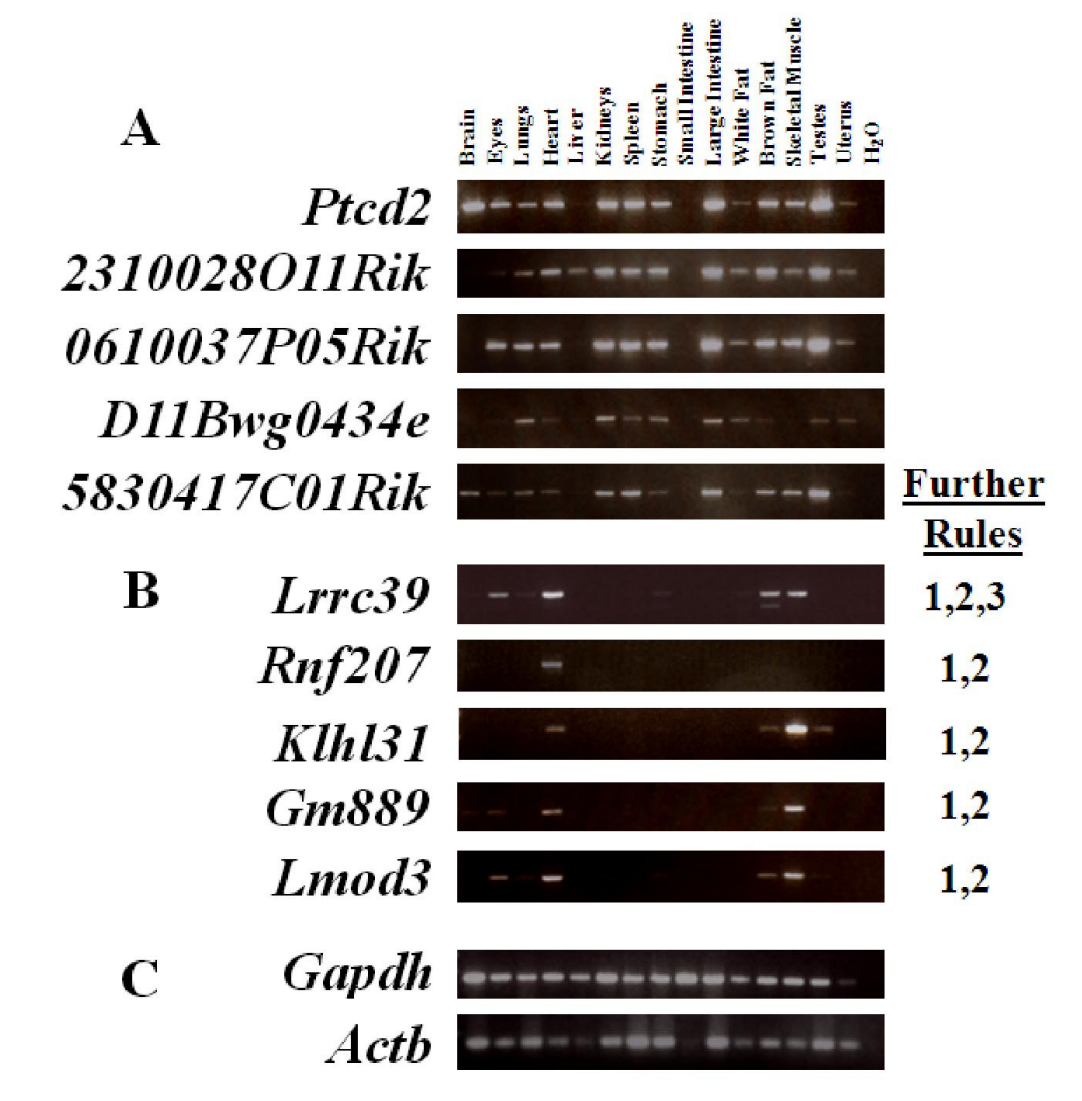
**RT-PCR results of selected genes**. RT-PCR results of candidate genes derived without (A) and with (B) inclusion of additional selections rules described in materials and methods. The selection rules, which were applied for each of the analyzed are shown in (B) next to the images of the agarose gels. (C) *Gapdh *and *Actb *were used as loading controls. Water was used as a negative control for all reactions. With the exception of *Gapdh *and *Actb *(25 cycles), 28 amplification cycles were used.

**Figure 4:**
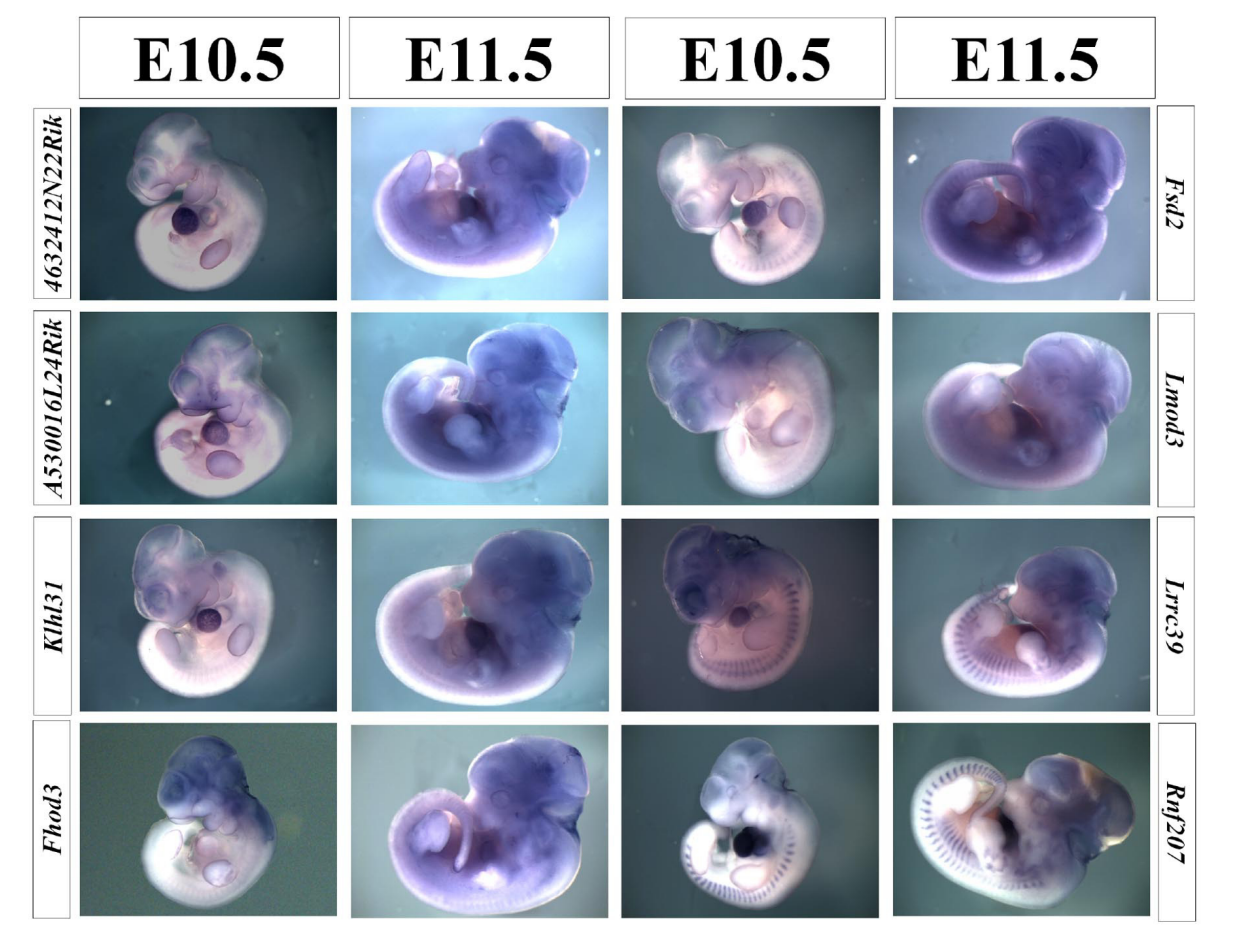
**WISH results of selected genes**. Expression patterns of 8 representative genes are shown. Forelimbs were removed from E11.5 embryos to reveal the expression in the heart, if necessary.

To validate the data obtained by our selection rules and to confirm the database records, RT-PCR experiments using mouse adult organs were performed. Surprisingly, we found that a large number of genes were expressed in multiple organs (Figure 3A). We therefore devised a set of additional rules to exclude ubiquitously expressed genes: (i) Genes must be expressed in less than 50% of total tissues; (ii) Sum of Gene EST must be less than 400; and (iii) At least 25% of Gene EST must be expressed in the target tissue (e.g. heart). Application of these rules efficiently eliminated ubiquitously expressed genes and left genes that were expressed preferentially in the heart (Figure 4B). Interestingly, most of the genes that remained after this selection were also expressed in striated muscles. This result was not unexpected given the fact that skeletal muscles and the heart are both composed of striated muscle cells, which share numerous morphological and physiological properties. Table 1 lists the number of mouse genes that were identified by using the combination of our selection rules. Inclusion of all three additional selection rules left only 30 mouse genes while inclusion of selection rules (i) and (ii) left 920 genes. As shown in Figure 4B, inclusion of selection rules (i) and (ii) did already efficiently exclude ubiquitously expressed genes. Addition of the third selection rule did not improve massively the number of genes with a heart-enriched expression pattern but result in a general decrease of selected genes. The inclusion of the third selection criterion proved to be helpful only for some cases (data not shown).

**Table 1:** Combination of selection rules successively restricts the number of heart-enriched genes

**Selection Category**	**Number of Mouse Genes**
Number of heart-enriched genes	2,348

With less than 2 publications in heart field in all 4 organisms	1,975

+ Mouse Further Rules 1 and others	926

+ Mouse Further Rules 1,2 or 1,2,3	920

+ Mouse Further Rules 1,2,3	30

The total numbers of genes that were identified using various combinations of selection tools are listed.

### Identification of Developmentally Expressed Genes

The expression in the adult heart does not necessarily mean that the corresponding gene is also expressed during early heart development. We therefore chose 20 genes from the genes that emerged from the computational screening and subjected them to whole-mount in situ hybridization (WISH) using E10.5 and E11.5 mouse embryos. At this stage, most organs have either formed or undergo organogenesis. 18 out of the 20 genes showed expression in the heart while 2 genes failed to display a specific expression at these stages (Figure 4 and data not shown). Based on the expression pattern, we distinguished two different groups: Group 1 showed equally strong expression in the heart both at E10.5 and E11.5 (*4632412N22Rik*, *A5530016L24Rik*, *Klhl31*, *Fsd2*, *Lrrc39*, and *Rnf207*); and Group 2 showed an increase of expression at E11.5 compared to E10.5 (*Fhod3 *and *Lmod3*).

### Preliminary Functional Characterization of Selected Genes by Loss-of-Function in Zebrafish Embryos

To obtain initial functional data for the selected genes, we turned to a loss-of-function analysis. Two uncharacterized, heart-enriched genes were chosen: leucine rich repeat containing 39 (*Lrrc39*) and kelch-like 31 (Drosophila) (*Klhl31*). *Lrrc39 *is highly expressed in the adult heart based on the RT-PCR analysis, and also found in eyes, skeletal muscle, brown fat, and weakly in the stomach (Figure 3B). During development, *Lrrc39 *is expressed at E10.5 in the heart, in somites and in the eye anlagen (Figure 4). *Klhl31 *showed a slightly different expression pattern. It was expressed in the adult heart, brown fat, skeletal muscle, and testes (Figure 3B). *Klhl31 *was also detected very strongly in the heart during development at E10.5 by WISH (Figure 4).

We decided to accomplish the loss-of-function analysis in zebrafish embryos because knock-down of genes is relatively easily achieved in zebrafish by injection of antisense morpholinos. In addition, the development of the cardiovascular system in zebrafish embryos can be conveniently monitored by microscopical inspections. The zebrafish homologues of *Lrrc39 *and *Klhl31 *were identified using HomoloGene [25] yielding "*zgc:112088*" and "*klhl31*" (which are indicated as "*zLrrc39*" and "*zKlhl31*" respectively). Morpholinos against "*zLrrc39*" and "*zKlhl31*" were directed at their ATG sites.

**Figure 5:**
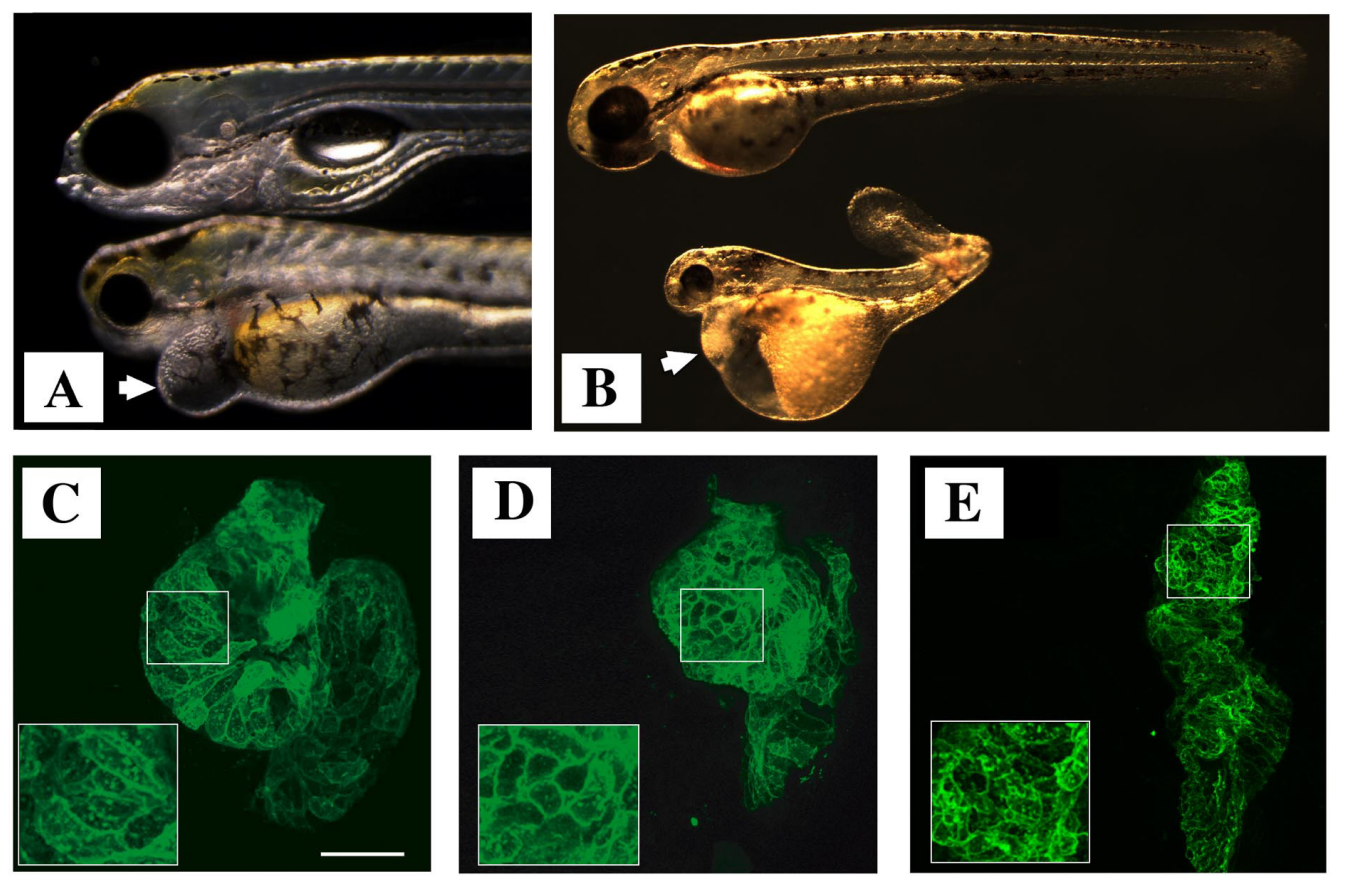
**Loss-of-function phenotypes after morpholino injections**. (A) Effect of *zLrrc39 *at 108 hpf and (B) *zKlhl31 *morpholino injections on zebrafish development at 48 hpf, respectively. Lateral views of non-injected control (upper) and morphant (lower) embryos (Tg(myl7:EGFP-HRAS)s883) are shown. The arrow in (A) indicates the pericardial edema in injected *zLrrc39 *morphant embryos. (C-E) Fluorescent images of heart sections at 48 hpf of control (C), *zLrrc39 *(D) and *zKlhl31 *(E) morphants are shown. Cardiomyocytes of zebrafish embryos were marked by expression of EGFP to delineate cellular morphology. Scale bar: 50 μm.

*zLrrc39 *morphants showed the first signs of disturbed development at 48 hour post fertilization (hpf). At 108 hpf, *zLrrc39 *morphants displayed a considerable pericardial edema, which is a characteristic sign of cardiac malfunctions (Figure 5A). All morphants died within 12 hours after this time point. To facilitate detection of developmental defects in the heart, we employed a transgenic zebrafish line "*Tg(myl7:EGFP-HRAS)*^*s*883^" [26], in which myocardial cells were labeled by GFP. Macroscopical evaluation of injected and control embryos at 48 hpf followed by sectioning revealed a disturbed arrangement of cardiomyocytes in the hearts of *zLrrc39 *morphants, but not in control embryos, indicating a role of *zLrrc39 *in morphogenetic processes controlling heart development (Figure 5B, C). In addition, *zLrrc39 *morphants were characterized by small eyes, malformations of the lower jaw, and distorted muscle structures (Figure 5A). It was interesting to note that the affected organs reflected the expression profile of *zLrrc39 *during mouse development.

The knock-down of *zKlhl31 *resulted in a more severe phenotype compared to *zLrrc39 *essentially abrogating development at 72 hpf. At 48 hpf, *zKlhl31 *morphants showed cardiac and yolk sac edema as well as a distorted body axis (Figure 5D). Sectioning of the heart of *zKlhl31 *morphants revealed that ventricle morphogenesis was severely disturbed, which essentially prevented looping of the heart.

## Discussion

In the current study, we have devised a new strategy to identify and characterize organ-specific gene, which lack a detailed functional annotation. We reason that the identification and characterization of such genes will facilitate future attempts to understand biological phenomena as a collection of interconnected systems. Our approach was based on a systematic exploitation of various databases such as UniGene. UniGene is a NCBI's database system for automatically partitioning GenBank sequences, including ESTs, into a non-redundant set of gene-oriented clusters [23]. Each UniGene cluster contains sequences that represent a unique gene or expressed pseudogene, together with related data sets including information about tissue types in which the gene is expressed, model organism protein similarities, and genomic locations. As of May 2008, 97 species and thousands of sequence libraries from various tissues were used to build UniGene clusters. In principle, UniGene contains comprehensive expression profiles based on sequencing results, which can be used to obtain information about expression patterns for a particular gene. UniGene also provides a category called "Restricted Expression". In order for a UniGene gene cluster to fall under this category, more than half of the GenBank sequences assigned to the cluster must come from the same source tissue. In Mus musculus, there are only 34 gene clusters (e.g. *Myh6*, *Myh7*, and *Nppa*) listed under "Restricted Expression" in "heart", which limits this application to genes that are not expressed in any other tissue and restricts the usefulness of this tool.

To overcome the loss-of-information, which occurs when applying such strict criteria, we developed selection rules that are based on the conservation of expression profiles of homologous genes between different species. This strategy successfully enlarged the number of detected genes without compromising the specificity of the detection.

Many studies have been conducted to identify tissue/organ-specific genes with known and unknown functions (reviewed extensively in [27]): endothelial [28]; epidiymis [29-31]; heart [32-35]; mammary gland [34]; pancreas [34]; preimplantation stages [36,37]; prostate [38]; skeletal muscle [39]; and testis [40]. Other studies focused on the discovery of biomarkers for diseases such as colon [41] and prostate cancer [42]. Most of these studies utilized cDNA or EST sequences and libraries from dbEST [43] or UniGene to screen for tissue/organ-specific genes. Some of these studies were validated by additional computational methods while others used RT-PCR or Northern blotting experiments to confirm the initial database searches. Only two studies included functional data [33,35]. In contrast to previous studies, which restricted the analysis to one or two species, we included four different organisms to identify species-conserved, heart-enriched expression patterns.

Several databases such as dbEST or UniGene [44-47] provide knowledge about tissue/organ-specific genes and give information about expression in different organisms [48] but are not particularly useful to serve as a starting point for further functional studies of uncharacterized genes. Our approach is simple and intuitive and does not require extensive programming and computational knowledge. We have demonstrated that DGSA (= Database-dependent Gene Selection and Analysis) provides an efficient means to select hitherto uncharacterized genes for further functional analysis. Since our selection criteria strongly relied on the conservation of expression profiles among species, it was straightforward to turn to a functional analysis of identified genes using non-amniotes model organisms such as zebrafish, which are particularly suited for rapid functional characterization using morpholino injections to achieve a loss-of-function phenotype. Selected genes might also be linked easily to databases of non-amniotes model organisms such as the Zebrafish Model Organisms Database (ZFIN) [49]. As of April 6, 2008, 89 zebrafish homologs of heart-enriched genes were included in this database (data not shown). Of these, 25 (corresponds to 28% coverage) were linked to phenotypes in heart or cardiac-related structures (e.g. cardioblast differentiation, cardiac ventricle). Further efforts to characterize mutants of genes, which were identified by DGSA in amniotes, will certainly increase this coverage in the future.

One might argue that a selection for conserved expression patterns might artificially restrict the number of genes, which can be detected or lead to the identification of genes that are not involved in the development, maintenance, or remodeling of the heart. To address this potential criticism, we matched the genes, which were identified by our selection criteria with GO terms for known heart-enriched genes. The fact that our algorithm provided 30% or more coverage for genes that are known to be involved in "heart development (GO:0007507)" clearly indicates that our selection rules work efficiently even without performing additional biological experiments. Although the current study focused on heart-enriched genes, we reasoned that our selection rules might be easily extended to other organs, such as brain, liver, spleen, and testis. In fact, we found that application of our selection rules to the above mentioned organs yielded the same coverage of GO terms as for the heart (Additional File 3 for Venn diagrams and Additional File 4 for GO coverage).

## Conclusion

We have introduced an efficient way to screen for "heart-enriched" genes with unknown functions. The computational approach to screen for genes across 4 species is simple and intuitive and might be applied by experimental biologists without programming knowledge. The presented strategy can be easily extended by implementing expression information about developing embryos obtained through public microarray and SAGE databases. Further functional analysis of identified genes will help to fill the gaps in our knowledge, which prevents a comprehensive understanding of complex molecular interactions as intended by "Systems Biology" researchers.

## Methods

In the following section, all dataset names are indicated by quotation marks.

### Selection Rules for UniGene Profiles

The data sources for all datasets used in this study are listed in Additional File 5 including the FTP sites, dates of data retrievals, and version numbers, if applicable. As a starting point, UniGene's "Mm.data" was used to search for the number of mouse GeneIDs (GI) with UniGene IDs. Next, HomoloGene's "homologene.data", was used to search for homologs and their UniGene IDs in rat (Rattus norvegicus; Taxonomy ID 10116), human (Homo sapiens; Taxonomy ID 9606), and chicken (Gallus gallus; Taxonomy ID 9031). The UniGene tissue expression profiles ("Mm.profiles" for mouse, "Rn.profiles" for rat, "Hs.profiles" for human, and "Gga.profiles" for chicken) were screened with the selection rule: "Select genes whose rank for 'heart' is under the top 20% across homologs." At the time of the screening, the number of tissues for each organism and the definition of the top 20% were as follows (organism name, number of tissues, number of tissues that fall under the top 20% category): Mouse, 47, 9; Rat, 25, 5; Human, 45, 9; and Chicken, 18, 4. Selected genes were subjected to the following further selection rules to eliminate ubiquitously expressed genes in mouse: (i) Expressed in less than 50% of total tissues. (ii) Sum of Gene EST must be less than 400. (iii) At least 25% of Gene EST must be expressed in the target tissue. A flowchart of the UniGene in silico screening procedure is given in Figure 1.

### Identification of Publications Corresponding to GIs

To find the number of publications related to an identified gene, Entrez Gene's "gene2pubmed" was used. First, the number of GIs that are listed under each PMID was counted and classified. Publications that include more than 100 GIs were considered as articles that report large screening results (e.g. microarrays, sequencing) and excluded to be counted for the number of publications for each gene. Next, PMIDs whose classification in Medical Subject Headings (MeSH) is under "heart" were searched and cross matched to the number of publications for each gene. If this number was less than 2, the GI (gene) was defined as being "uncharacterized".

### Searching for Genes with GO Terms Related to Heart

Gene Ontology [24] terms for "heart" were searched through AmiGO [50]. Next, using these GO terms, gene names and their symbols were extracted from Entrez Gene's "gene2go".

To demonstrate the statistical significance of GO coverage, Fisher's exact test was applied. First, numbers of genes with at least one GO term were computed from Entrez Gene's "gene2go", which resulted in 18,225 genes for mouse, 16,437 for rat, 17,905 for human, and 9,226 for chicken. Then, a Fisher's exact test was applied to 2,348 heart-enriched genes for each GO term and its coverage.

### RNA Preparation, First-Strand cDNA Synthesis and RT-PCR

To extract total RNA, adult mice were sacrificed, and washed extensively with ice-cold PBS to remove blood. Isolated organ parts were frozen in liquid nitrogen and homogenized using TRIzol Reagent (Invitrogen). 2 μg of purified RNA was reverse transcribed using SuperScript II First-Strand Synthesis System (Invitrogen) to synthesize the first-strand cDNA by following the manufacturer's protocol. First-strand cDNA was diluted with water to generate a 1:5 dilution. 1 μl of cDNA was used for each PCR reaction. The following thermal profile was used for all PCR experiments: 95°C for 2 minutes, denaturation (94°C) for 30 seconds, annealing (60–64°C) for 30 seconds, extension (72°C) for 1 minute, followed by a final extension (72°C) for 7 minutes. Amplified PCR products were separated in 2% agarose gel and visualized using UV light.

### Whole-Mount In Situ Hybridization

To generate RNA probes, PCR amplified products were purified from the gel and cloned using pGEM-T Easy System (Promega). Antisense RNA probes were labeled with digoxigenin-UTP (Roche), using SP6 or T7 RNA polymerase (Promega). Embryos were fixed in 4% paraformaldehyde (PFA) dissolved in PBS/DEPC-treated water overnight and dehydrated the next day with 25%, 50%, 75%, and 100% methanol. For whole-mount in situ hybridizations, published protocols were used [51] with minor modifications. After staining, embryos were post-fixed in 4% PFA and stored at 4°C until photographs were taken.

### Morpholino Injection

The zebrafish strains used in this study were "Bad Nauheim (BNA)", which was originated from a local pet shop and was inbred for several generations, and "*Tg(myl7:EGFP-HRAS)*^*s*883^" [25]. Antisense morpholinos were purchased from BioCat (Clone ID MORPH 1769 for *zLrrc39*) and GeneTools (*zKlhl31*). For the morpholino experiments, 3 concentrations were tested (approximately 2–4 nl of morpholino diluted in sterile water/phenol red as an indicator per zebrafish egg): "1:2 dilution (high)", which corresponds to 8.5–17 ng of morpholino; "1:5 dilution (medium)", 3.4–6.8 ng; and "1:10 dilution (low)", 1.7–3.4 ng. For each injection, more than 50 eggs were injected. As a control, sterile water/phenol red without morpholino was used to observe effects caused by the injection.

## Authors' contributions

SU conceived the study, performed computational studies, contributed to the molecular genetics studies, and drafted the manuscript. AU conceived the study and contributed to the molecular genetics studies. MW carried out morpholino injections. BJ carried out morpholino injections and analyzed zebrafish phenotypes. PZ contributed to the molecular genetic studies. KJ analyzed zebrafish phenotypes. KgK and WS participated in the design of the study. TB participated in the design and coordination of the study and wrote the final version of the manuscript. All authors read and approved the final manuscript.

## Supplementary Material

Additional file 1 **Coverage of GO terms by genes that matched the applied selection criteria.** For each organism, the percentage coverage was derived by dividing the number of genes obtained (numerator in the parenthesis) by the number of genes that are classified under a specific GO term (denominator in the parenthesis). Fisher's exact test was applied to each GO coverage. Click here for file

Additional file 2 **Combinatorial GO annotations of mouse genes categorized under "heart development". **43 genes that are categorized under "heart development (GO:0007507)" were obtained. Gene symbols, GeneIDs, and combinations of organisms, in which they were selected, are indicated. Click here for file

Additional file 3 **Venn diagrams for brain-, liver-, spleen-, and testis-enriched genes.** The numbers of tissue-enriched genes selected for the top 20% in the ranking of UniGene tissue expression profiles are shown for the 4 organisms analyzed and displayed as combinations of these organisms. In each combination, the number of genes with less than 2 articles after MeSH term filtering for the corresponding tissue is shown in parenthesis. (A) brain-enriched genes; (B) liver-enriched genes; (C) spleen-enriched genes; and (D) testis-enriched genes. Click here for file

Additional file 4 **GO coverage for brain-, liver-, and spleen-enriched genes.** For each organism, the percentage coverage of the GO term for "development" of the corresponding tissue was derived by dividing the number of genes obtained (numerator in the parenthesis) by the number of genes that are classified under a specific GO term (denominator in the parenthesis). Fisher's exact test was applied to each GO coverage. In the case of "testis-enriched genes", there is no GO term for "testis development". Click here for file

Additional file 5 **List of datasets used in this study.** List of datasets used in this study. Click here for file
