# Supplementary material for: An integrated approach for the systematic identification and characterization of heart-enriched genes with unknown functions
Source: BMC Genomics. 2009 Mar 6;10:100. doi: 10.1186/1471-2164-10-100 (PMC2657154; doi:10.1186/1471-2164-10-100)
Supplement: Additional file 1 — Coverage of GO terms by genes that matched the applied selection criteria. For each organism, the percentage coverage was derived by dividing the number of genes obtained (numerator in the parenthesis) by the number of genes that are classified under a specific GO term (denominator in the parenthesis). Fisher's exact test was applied to each GO coverage. [file 1471-2164-10-100-S1.pdf]

GO ID	GO Term	Mouse	p-value (Mouse)	Rat	p-value (Rat)	Human	p-value (Human)	Chicken	p-value (Chicken)
GO:0007507	heart development	34% (43/124)	2,30E-07	28% (27/94)	0,0007661	37% (23/61)	4,58E-05	37% (6/16)	0,03272
GO:0003007	heart morphogenesis	36% (8/22)	0,01662	0% (0/3)	1	37% (3/8)	0,1219	0% (0/0)	1
GO:0008016	regulation of heart contraction	40% (8/20)	0,01084	37% (11/29)	0,004159	55% (15/27)	3,37E-05	0% (0/1)	1
GO:0001947	heart looping	35% (5/14)	0,05724	40% (4/10)	0,06645	33% (3/9)	0,1494	33% (1/3)	0,3842
GO:0035050	embryonic heart tube development	45% (5/11)	0,02864	33% (2/6)	0,2298	42% (3/7)	0,09652	66% (2/3)	0,103
GO:0007512	adult heart development	70% (7/10)	0,00172	77% (7/9)	0,001122	57% (4/7)	0,02887	66% (2/3)	0,103
GO:0002026	regulation of the force of heart contraction	50% (5/10)	0,02168	50% (3/6)	0,07361	40% (2/5)	0,1857	0% (0/1)	1
GO:0048738	cardiac muscle development	71% (5/7)	0,007652	100% (3/3)	0,02275	50% (3/6)	0,07361	66% (2/3)	0,103
GO:0060048	cardiac muscle contraction	57% (4/7)	0,02887	88% (8/9)	0,0002668	66% (2/3)	0,103	0% (0/0)	1
GO:0002027	regulation of heart rate	33% (2/6)	0,2298	50% (2/4)	0,1431	66% (2/3)	0,103	0% (0/0)	1
GO:0055007	cardiac muscle cell differentiation	60% (3/5)	0,0535	66% (2/3)	0,103	66% (2/3)	0,103	100% (1/1)	0,2152
GO:0055010	ventricular cardiac muscle morphogenesis	60% (3/5)	0,0535	83% (5/6)	0,004922	71% (5/7)	0,007652	0% (0/0)	1
GO:0060038	cardiac muscle cell proliferation	40% (2/5)	0,1857	0% (0/1)	1	50% (2/4)	0,1431	0% (0/1)	1
GO:0055003	cardiac myofibril assembly	100% (4/4)	0,008105	66% (2/3)	0,103	100% (1/1)	0,2152	100% (2/2)	0,008105
GO:0045822	negative regulation of heart contraction	50% (2/4)	0,1431	40% (2/5)	0,1857	50% (1/2)	0,3048	0% (0/1)	1
GO:0055012	ventricular cardiac muscle cell differentiation	0% (0/4)	1	0% (0/1)	1	0% (0/0)	1	0% (0/0)	1
GO:0001996	positive regulation of heart rate by epinephrine-norepinephrine	66% (2/3)	0,103	66% (2/3)	0,103	50% (1/2)	0,3048	0% (0/0)	1
GO:0001997	positive regulation of the force of heart contraction by epinephrine-norepinephrine	66% (2/3)	0,103	66% (2/3)	0,103	50% (1/2)	0,3048	0% (0/0)	1
GO:0060047	heart contraction	66% (2/3)	0,103	100% (1/1)	0,2152	100% (2/2)	0,06675	0% (0/0)	1
GO:0035051	cardiac cell differentiation	33% (1/3)	0,3842	50% (1/2)	0,3048	100% (1/1)	0,2152	0% (0/0)	1
GO:0060045	positive regulation of cardiac muscle cell proliferation	0% (0/3)	1	0% (0/1)	1	0% (0/1)	1	0% (0/0)	1
GO:0048739	cardiac muscle fiber development	100% (2/2)	0,06675	100% (1/1)	0,2152	100% (1/1)	0,2152	100% (1/1)	0,2152
GO:0035054	embryonic heart tube anterior/posterior pattern formation	50% (1/2)	0,3048	50% (1/2)	0,3048	0% (0/0)	1	100% (1/1)	0,2152
GO:0055015	ventricular cardiac muscle cell development	50% (1/2)	0,3048	100% (1/1)	0,2152	0% (0/0)	1	0% (0/0)	1
GO:0001985	negative regulation of heart rate in baroreceptor response to increased systemic arterial blood pressure	100% (1/1)	0,2152	100% (1/1)	0,2152	0% (0/0)	1	0% (0/0)	1
GO:0055008	cardiac muscle morphogensis	100% (1/1)	0,2152	50% (1/2)	0,3048	100% (1/1)	0,2152	100% (2/2)	0,06675
GO:0055009	atrial cardiac muscle morphogenesis	100% (1/1)	0,2152	100% (1/1)	0,2152	100% (1/1)	0,2152	100% (1/1)	0,2152
GO:0055013	cardiac muscle cell development	100% (1/1)	0,2152	0% (0/0)	1	0% (0/0)	1	0% (0/0)	1
GO:0001986	negative regulation of the force of heart contraction during baroreceptor response to increased systemic arterial blood pressure	0% (0/1)	1	0% (0/1)	1	0% (0/1)	1	0% (0/0)	1
GO:0001988	positive regulation of heart rate in baroreceptor response to decreased systemic arterial blood pressure	0% (0/1)	1	0% (0/0)	1	0% (0/1)	1	0% (0/1)	1
GO:0045823	positive regulation of heart contraction	0% (0/1)	1	0% (0/2)	1	0% (0/2)	1	0% (0/1)	1
GO:0060044	negative regulation of cardiac muscle cell proliferation	0% (0/0)	1	50% (1/2)	0,3048	100% (1/1)	0,2152	100% (1/1)	0,2152
