# Supplementary material for: An integrated approach for the systematic identification and characterization of heart-enriched genes with unknown functions
Source: BMC Genomics. 2009 Mar 6;10:100. doi: 10.1186/1471-2164-10-100 (PMC2657154; doi:10.1186/1471-2164-10-100)
Supplement: Additional file 2 — Combinatorial GO annotations of mouse genes categorized under "heart development". 43 genes that are categorized under "heart development (GO:0007507)" were obtained. Gene symbols, GeneIDs, and combinations of organisms, in which they were selected, are indicated. [file 1471-2164-10-100-S2.pdf]

Symbol	Mouse GeneID	Mouse Rat	Mouse Human	Mouse Chic	Rat Human	Rat Chick	Human Chick	Mouse Rat Human	Mouse Rat Chick	Mouse Human Chick	Rat Human Chick	Mouse Rat Human Chick
Acadm	11364	Yes	Yes	No	Yes	No	No	Yes	No	No	No	No
Acvr1	11477	No	Yes	No	No	No	No	No	No	No	No	No
Acvr2b	11481	No	Yes	No	No	No	No	No	No	No	No	No
Adam19	11492	No	No	No	Yes	No	No	No	No	No	No	No
Alpk3	116904	Yes	Yes	Yes	Yes	Yes	Yes	Yes	Yes	Yes	Yes	Yes
Bmp10	12154	Yes	No	No	No	No	No	No	No	No	No	No
Smyd1	12180	Yes	Yes	Yes	Yes	Yes	Yes	Yes	Yes	Yes	Yes	Yes
Casq2	12373	Yes	Yes	Yes	Yes	Yes	Yes	Yes	Yes	Yes	Yes	Yes
Vcan	13003	No	No	No	Yes	No	No	No	No	No	No	No
Cxadr	13052	No	No	No	No	Yes	No	No	No	No	No	No
Ednra	13617	Yes	Yes	Yes	Yes	Yes	Yes	Yes	Yes	Yes	Yes	Yes
Eng	13805	No	Yes	No	No	No	No	No	No	No	No	No
Erbb2	13866	Yes	No	No	No	No	No	No	No	No	No	No
Gata4	14463	Yes	Yes	No	Yes	No	No	Yes	No	No	No	No
Gja7	14615	No	Yes	No	No	No	No	No	No	No	No	No
Gli2	14633	No	No	No	Yes	No	No	No	No	No	No	No
Gli3	14634	No	No	No	No	No	Yes	No	No	No	No	No
Gys1	14936	Yes	No	No	No	No	No	No	No	No	No	No
Hand1	15110	Yes	No	No	No	No	No	No	No	No	No	No
Hand2	15111	No	No	No	No	Yes	No	No	No	No	No	No
Id2	15902	No	No	No	No	No	Yes	No	No	No	No	No
Kcnj8	16523	No	Yes	No	No	No	No	No	No	No	No	No
Mb	17189	Yes	Yes	Yes	Yes	Yes	Yes	Yes	Yes	Yes	Yes	Yes
Mef2c	17260	No	Yes	No	No	No	No	No	No	No	No	No
Foxc1	17300	No	Yes	No	No	No	No	No	No	No	No	No
Msx2	17702	No	No	No	Yes	No	No	No	No	No	No	No
Myl2	17906	Yes	Yes	Yes	Yes	Yes	Yes	Yes	Yes	Yes	Yes	Yes
Nf1	18015	No	No	Yes	No	No	No	No	No	No	No	No
Nfatc1	18018	No	Yes	No	No	No	No	No	No	No	No	No
Nkx2-5	18091	Yes	Yes	No	Yes	No	No	Yes	No	No	No	No
Nrp1	18186	No	No	Yes	No	No	No	No	No	No	No	No
Srf	20807	Yes	No	No	No	No	No	No	No	No	No	No
Tbx5	21388	No	Yes	Yes	No	No	Yes	No	No	Yes	No	No
Myocd	214384	Yes	Yes	No	Yes	No	No	Yes	No	No	No	No
Tead1	21676	No	No	No	Yes	No	No	No	No	No	No	No
Tnni3	21954	Yes	No	No	No	No	No	No	No	No	No	No
Tnnt2	21956	Yes	Yes	Yes	Yes	Yes	Yes	Yes	Yes	Yes	Yes	Yes
Mib1	225164	No	Yes	No	No	No	No	No	No	No	No	No
Zfpm2	22762	No	No	No	Yes	No	No	No	No	No	No	No
Txnrd2	26462	Yes	No	Yes	No	Yes	No	No	Yes	No	No	No
Irx4	50916	Yes	No	Yes	No	Yes	No	No	Yes	No	No	No
Pkp2	67451	Yes	Yes	Yes	Yes	Yes	Yes	Yes	Yes	Yes	Yes	Yes
Hopx	74318	No	No	Yes	No	No	No	No	No	No	No	No
