# Supplementary figures and images for: An integrated approach for the systematic identification and characterization of heart-enriched genes with unknown functions
Source: BMC Genomics. 2009 Mar 6;10:100. doi: 10.1186/1471-2164-10-100 (PMC2657154; doi:10.1186/1471-2164-10-100)

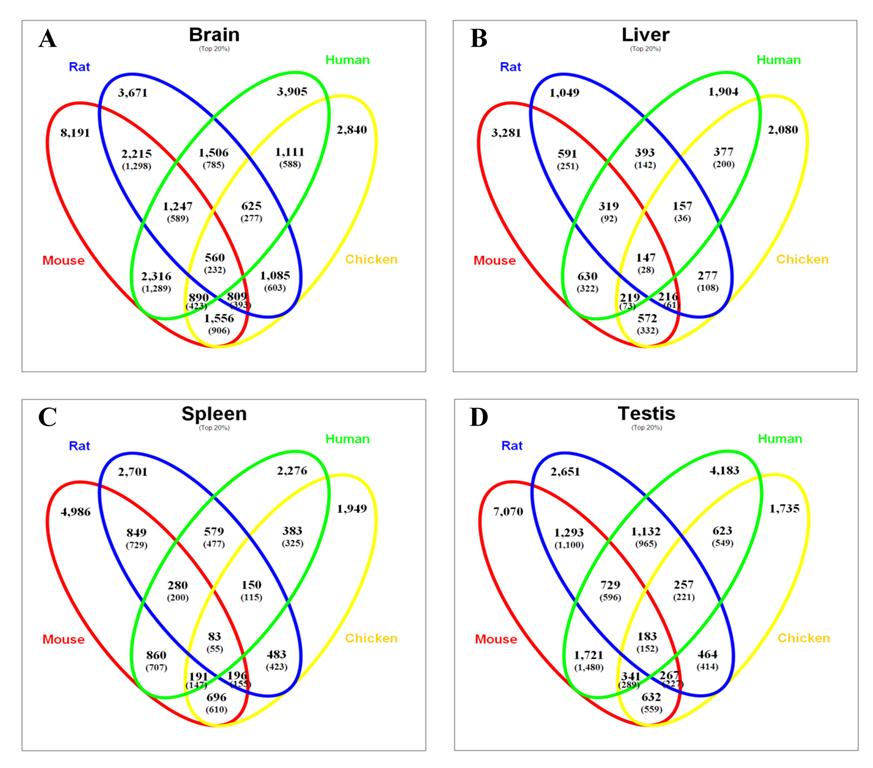

Supplement: Additional file 3 — Venn diagrams for brain-, liver-, spleen-, and testis-enriched genes. The numbers of tissue-enriched genes selected for the top 20% in the ranking of UniGene tissue expression profiles are shown for the 4 organisms analyzed and displayed as combinations of these organisms. In each combination, the number of genes with less than 2 articles after MeSH term filtering for the corresponding tissue is shown in parenthesis. (A) brain-enriched genes; (B) liver-enriched genes; (C) spleen-enriched genes; and (D) testis-enriched genes. [file 1471-2164-10-100-S3.jpeg]
