# Supplementary material for: An integrated approach for the systematic identification and characterization of heart-enriched genes with unknown functions
Source: BMC Genomics. 2009 Mar 6;10:100. doi: 10.1186/1471-2164-10-100 (PMC2657154; doi:10.1186/1471-2164-10-100)
Supplement: Additional file 4 — GO coverage for brain-, liver-, and spleen-enriched genes. For each organism, the percentage coverage of the GO term for "development" of the corresponding tissue was derived by dividing the number of genes obtained (numerator in the parenthesis) by the number of genes that are classified under a specific GO term (denominator in the parenthesis). Fisher's exact test was applied to each GO coverage. In the case of "testis-enriched genes", there is no GO term for "testis development". [file 1471-2164-10-100-S4.pdf]

GO ID	GO Term	Mouse	p-value (Mouse)	Rat	p-value (Rat)	Human	p-value (Human)	Chicken	p-value (Chicken)
GO:0007420	brain development	46% (29/62)	1,53E-07	48% (40/83)	3,67E-10	52% (39/74)	8,13E-11	37% (3/8)	0,1219
GO:0001889	liver development	33% (7/21)	0,03423	45% (9/20)	0,003845	40% (4/10)	0,06645	50% (2/4)	0,1431
GO:0048536	spleen development	33% (3/9)	0,1494	0% (0/1)	1	75% (3/4)	3,65E-02	50% (1/2)	0,3048
