# Supplementary material for: An integrated approach for the systematic identification and characterization of heart-enriched genes with unknown functions
Source: BMC Genomics. 2009 Mar 6;10:100. doi: 10.1186/1471-2164-10-100 (PMC2657154; doi:10.1186/1471-2164-10-100)
Supplement: Additional file 5 — List of datasets used in this study. List of datasets used in this study. [file 1471-2164-10-100-S5.pdf]

Database Name	FTP Site	Date of Data Retrieval	Note (e.g. version)
Entrez Gene	ftp://ftp.ncbi.nih.gov/gene/	10/4/2007	
HomoloGene	ftp://ftp.ncbi.nih.gov/pub/HomoloGene/	10/4/2007	build57
Mouse Genome Informatics (MGI)	ftp://ftp.informatics.jax.org/	10/9/2007	
UniGene	ftp://ftp.ncbi.nih.gov/repository/UniGene/	10/4/2007	
Medical Subject Headings (MeSH)	http://www.ncbi.nlm.nih.gov/sites/entrez?db=mesh	12/8/2007	
